# A Multimodal Perspective on the Composition of Cortical Oscillations

**DOI:** 10.3389/fnhum.2013.00132

**Published:** 2013-04-10

**Authors:** Kim C. Ronnqvist, Craig J. McAllister, Gavin L. Woodhall, Ian M. Stanford, Stephen D. Hall

**Affiliations:** ^1^Aston Brain Centre, School of Life and Health Sciences, Aston UniversityBirmingham, UK; ^2^Faculty of Science and Technology, School of Psychology, Plymouth UniversityDevon, UK

**Keywords:** motor cortex, magnetoencephalography, local field potential, oscillation, gamma-aminobutyric acid, beta rhythm, mu rhythm, BOLD fMRI

## Abstract

An expanding corpus of research details the relationship between functional magnetic resonance imaging (fMRI) measures and neuronal network oscillations. Typically, integrated electroencephalography and fMRI, or parallel magnetoencephalography (MEG) and fMRI are used to draw inference about the consanguinity of BOLD and electrical measurements. However, there is a relative dearth of information about the relationship between E/MEG and the focal networks from which these signals emanate. Consequently, the genesis and composition of E/MEG oscillations requires further clarification. Here we aim to contribute to understanding through a series of parallel measurements of primary motor cortex (M1) oscillations, using human MEG and *in vitro* rodent local field potentials. We compare spontaneous activity in the ∼10 Hz mu and 15–30 Hz beta frequency ranges and compare MEG signals with independent and integrated layers III and V (LIII/LV) from *in vitro* recordings. We explore the mechanisms of oscillatory generation, using specific pharmacological modulation with the GABA-A alpha-1 subunit modulator zolpidem. Finally, to determine the contribution of cortico-cortical connectivity, we recorded *in vitro* M1, during an incision to sever lateral connections between M1 and S1 cortices. We demonstrate that frequency distribution of MEG signals appear have closer statistically similarity with signals from integrated rather than independent LIII/LV laminae. GABAergic modulation in both modalities elicited comparable changes in the power of the beta band. Finally, cortico-cortical connectivity in sensorimotor cortex (SMC) appears to directly influence the power of the mu rhythm in LIII. These findings suggest that the MEG signal is an amalgam of outputs from LIII and LV, that multiple frequencies can arise from the same cortical area and that *in vitro* and MEG M1 oscillations are driven by comparable mechanisms. Finally, cortico-cortical connectivity is reflected in the power of the SMC mu rhythm.

## Introduction

The development of sophisticated non-invasive human neuroimaging methods has revolutionized our approach to understanding the function of the brain. In particular, the ubiquitous magnetic resonance imaging (MRI), capable of inferring the profile of neural activity from blood oxygen-level dependent (BOLD) measurements, has facilitated the rapid construction of a detailed functional map of the human brain. However, as a result of the indirect nature and temporal resolution of these measurements, it is important that further comparisons with direct electrophysiological measures are made, in order to properly extrapolate the information contained within the wealth of existing literature. For more than a decade, researchers have integrated functional magnetic resonance imaging (fMRI) information with electrophysiological measures such as magnetoencephalography (MEG), in order to improve the outcomes of these approaches (Liu et al., 1998; Dale et al., 2000; Dale and Halgren, 2001).

Early work to understand the neural basis of fMRI, focused on the comparison between MEG evoked responses and fMRI BOLD (e.g., Ahlfors et al., 1999; Dale et al., 2000). However, the advent of contemporary MEG approaches, such as beamforming, enabled the localization and reconstruction of oscillatory activity in the cortex with greatly improved spatial resolution (Vrba and Robinson, 2001; Hillebrand et al., 2005). With a focus on functionally related changes, these approaches have enabled researchers to explore the relationship between rhythmic signature of proposed importance in neural processing and the corresponding BOLD response. Parallel experiments using both language and visual tasks revealed a close correspondence between the oscillatory signatures of MEG and BOLD (Singh et al., 2002). Further studies have revealed the complex nature of these relationships, by exploring multiple oscillatory signatures in comparison with fMRI BOLD activity. Brookes et al. (2005), using a visual paradigm, demonstrated a positive correlation between BOLD and sustained fields and gamma (>30 Hz) power and a negative correlation between BOLD and alpha power; these observations are supported by a subsequent study that reported differential effects at low and high frequencies (Zumer et al., 2010). A number of further studies have since described observations of correspondence between MEG oscillations and fMRI BOLD (for a review see Singh, 2012). However, while these studies describe strong support for the comparative identification of the involvement of specific brain regions in specific function, the correspondence between modalities is not always as clear-cut. Examples from visual experiments, where gamma oscillations are implicated in the processing of features such as contrast (Hall et al., 2005) and spatial frequency (Adjamian et al., 2004), reveal discrepancies between the processing of color stimuli with MEG (Adjamian et al., 2008) compared to BOLD (Mullen et al., 2007). Similarly, the tuning characteristics for spatial frequency appear different between these modalities (Muthukumaraswamy and Singh, 2008, 2009). In recent years, the importance of understanding the natural variability of neuronal network activity at rest, has become a topic of great interest. Accordingly, so-called “resting state” networks have been investigated using fMRI approaches and recent studies have applied parallel fMRI and MEG to determine the oscillatory counterparts to the fMRI observations (Brookes et al., 2011).

The relationship between neuronal network oscillations and the BOLD response is further described in comparisons between BOLD signatures and invasive recordings in animals, where BOLD appears to temporally correlate well with the local field potential (LFP), for example in the gamma range (Logothetis et al., 2001). However, while these relationships provide, arguably, a more direct reflection of the neuronal network activity underlying the BOLD signal, they begin to reveal the additional complexities that exist in relating these signals. For example, experiments in the rat somatosensory cortex reveal correspondence between the BOLD signal and LFP oscillatory activity. However, the use of multi-channel depth electrodes reveals a depth dependent response to stimulation, demonstrating differential relationships between neuronal oscillations and the BOLD response (Boorman et al., 2010). The simultaneous recording of LFP activity during fMRI in the human auditory cortex reveals that the BOLD response is closely related to the gamma activity and, importantly, the interneuron activity at the same location (Nir et al., 2007).

With regard to the aim of understanding the neural basis of the fMRI signal. High spatial and temporal resolution, non-invasive measures of human brain function such as MEG, are powerful measures for understanding the electrophysiological basis of fMRI. However, the limitations in our understanding of the physiological and pharmacological basis of the MEG signal, limit the extent to which we can draw conclusions about the basis of the fMRI signal.

Previous studies have developed models of the neuronal population that form the generators of the MEG signal, based upon the cortical architecture and cell physiology (for a review see Hamalainen, 1992). Further studies suggest that the MEG signal is an aggregate of 50000 (perhaps as few as 10000) synchronously firing pyramidal cells in layer three (LIII) and layer five (LV) of the cortex (Murakami and Okada, 2006). This is particularly convenient for the purpose of comparison with oscillations *in vitro*, as we know that these same populations of cells give rise to the spontaneous oscillatory activity. These oscillations are driven by phasic inhibition of principal cells by interneurons and are well described in both modeling studies (Traub et al., 1996) and empirical data (Whittington et al., 1995). These oscillations in the cortex, for example the sensorimotor cortex (SMC), are driven largely by GABAergic interneurons (Roopun et al., 2006; Yamawaki et al., 2008). Furthermore, experimental evidence from MEG suggests that these are the same oscillations that are measurable in the MEG signal (Jensen et al., 2005; Hall et al., 2010a,b, 2011; Muthukumaraswamy et al., 2012). Further support for this comes from parallel MEG and magnetic resonance spectroscopy (MRS) studies that suggest that GABA concentration corresponds with oscillatory characteristics in various regions of the cortex (Muthukumaraswamy et al., 2009; Gaetz et al., 2011).

However, while these aspects appear to provide a strong basis for understanding the MEG signal, MEG measurement is such that a “virtual electrode” signal integrates the activity of >2 mm of cortical tissue (Barnes et al., 2004). Therefore, cortical signals measured with MEG will be an amalgam of signals from all cortical laminae in that location. In addition, as magnetic field strength decays with distance, the relative contributions of activity from superficial and deep cortical layers are unlikely to be equal. While the distance from sensor to cortical surface is undoubtedly greater than the distance between laminae, cortical thickness can be as great as 4.5 mm (Fischl and Dale, 2000) and therefore worthy of consideration.

As discussed, the predominant mechanism for ongoing oscillations are believed to be local interneuron generated activity. However, the extensive connectivity between the cortex and other cortical and sub-cortical structures results in the potential for great complexity in the generation of oscillations. For example, lower frequency oscillations, such as the alpha rhythm (7–14 Hz) are often believed to be an emergent property of thalamo-cortical loops (Rosanova et al., 2009). Furthermore, specific oscillations are often believed to originate from specific areas. For example, in the SMC the beta rhythm (15–30 Hz) is believed to arise in the primary motor cortex (M1) (Murthy and Fetz, 1992; Baker et al., 1997), whilst the mu rhythm (10 Hz) is believed to originate in the primary somatosensory cortex (S1) (Salmelin and Hari, 1994). However, it is uncertain to what extent each of the measured oscillatory signatures are inter-cortical (within cortical region) and which are intra-cortical (between regions) dependent.

In order to understand the relevance of the comparisons between fMRI and MEG, it is important to understand the relationship between the MEG signal and the underlying neuronal network activity. In this paper, we aim to contribute to the understanding of this question, using parallel *in vitro* and MEG measurements of M1 to characterize the relationship between the aggregate MEG signal and the underlying cortical tissue.

We focus on three simple aspects: (1) The relative contributions of oscillations arising in superficial (LIII) and deep (LV) cortical laminae to the MEG signal. (2) The comparative mechanisms of oscillations observed *in vitro* and with MEG. (3) The influence of cortico-cortical connectivity on the oscillations observed *in vitro* from M1.

## Materials and Methods

### Data acquisition

In each experiment, the focus for comparison was M1. The process for localizing M1 with MEG and preparing the M1 slice *in vitro* was identical in each experiment; these processes are described in the following sections.

### MEG

**Figure 1:**
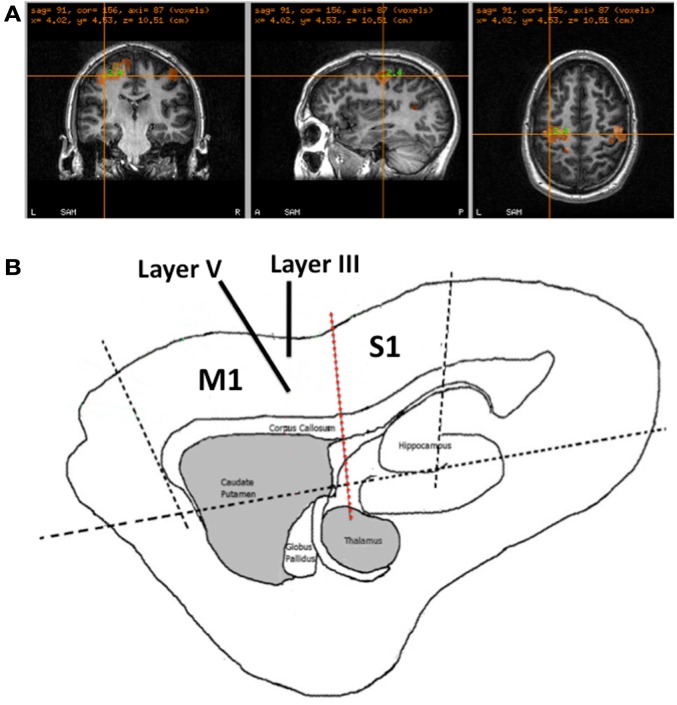
**Recording Methods for MEG and *in vitro***. **(A)** Example of the M1 localization in a participant based upon the identification of the PMBR response following movement. **(B)** Schematic of a sagittal rat brain slice, showing the location of M1 and S1. Black dotted line show where cuts were made in preparation for all recordings. Solid black lines show the positioning of the microelectrodes in the slice for recording LIII and LV LFPs and the red dashed line show the position of the cut made between M1 and S1 in experiment 3.

In each experiment, participants with normal or corrected to normal vision, were seated in a 275-channel MEG system (CTF Systems, Canada). MEG data were acquired at a sampling rate of 1200 Hz using a third order gradiometer configuration with a 50-Hz notch filter and a 1–300-Hz low/high pass filter. MEG data were co-registered with the individual participant’s anatomical MRI, obtained using a 3-Tesla MRI system (Siemens, Erlangen, Germany), by surface matching a three-dimensional digitization of the participants scalp created using a Polhemus Isotrak system (Kaiser Aerospace Inc.). Head position was monitored throughout by matching the digitized position of three surface-mounted electromagnetic positioning coils (left and right pre-auricular and nasion), which were then monitored throughout the recording process. In each experiment, participants performed a series of 60 left and right index finger abduction movements, approximately every 6 s; finger movements were monitored using electromyography (EMG) of the first dorsal interosseus muscle. Here the left M1 cortex was localized (Figure 1A) using the synthetic aperture magnetometry (SAM) beamforming method (Vrba and Robinson, 2001; Hillebrand et al., 2005). Specifically, with time-zero defined as the offset in EMG power following movement, defined as a reduction below three standard-deviations of the baseline, the post-movement beta rebound (PMBR) was localized by comparing the change in beta (15–30 Hz) frequency power following movement termination (0.5–1.0 s) with the pre-movement beta power (−2.0 to −1.5 s), comparable to the methods described by Jurkiewicz et al. (2006). In each MEG experiment, the envelope of neuronal network activity in M1 was reconstructed during a period of inactive rest, using the virtual electrode method previously described (Hall et al., 2010a, 2011).

### In vitro

Recordings were made in sagittal brain slices obtained from p18 to p22 male Wistar rats (40–60 g), 450 μm thick, containing M1 and S1 (Figure 1B). All experiments were performed in accordance with the Animals (Scientific Procedures) Act 1986, European Communities Directive 1986 (86/609/EEC) and the Aston University ethical review documents. Every effort was made to minimize the number of animals used and their suffering. Animals were anesthetized with isoflurane and, following decapitation, the brain removed and placed in modified artificial cerebrospinal fluid (aCSF). The aCSF solution was composed with the recipe (in mM): 206 sucrose, 2 KCl, 1.6 MgSO_4_, 25.9 NaHCO_3_, 1.25 NaH_2_PO_4_, 10 glucose, 2.16 CaCl_2_, and indomethacin 45 nM (Pakhotin et al., 1997), saturated with 95% O_2_ and 5% CO_2_ at pH 7.3 and 310 mOsm. Brain slices were prepared using a microslicer (Campden Instruments, Loughborough, UK) and, prior to recording, maintained at room temperature in aCSF containing (in mM): 126 NaCl, 4.0 KCl, 0.5 MgSO_4_, 25.9 NaHCO_3_, 1.25 NaH_2_PO_4_, 10 glucose, and 2 CaCl_2_ for at least 60 min. For recording, slices were placed in an interface recording chamber (Scientific System Design Inc., Canada) and superfused with aCSF at a flow rate of 2 ml/min maintained at 34°C. Spontaneous oscillatory activity was restored using the addition of carbachol (50 μm) and kainic acid (400 nM) (Sigma Ltd., Gillingham, UK) as previously described (Yamawaki et al., 2008). Extracellular LFP recordings were made with electrodes constructed of chlorided silver wire inserted into a borosilicate glass electrode filled with aCSF of resistance 1–3 MΩ. The glass electrodes, attached to micro-manipulators (Narashige, Japan) were inserted in LIII and LV of the M1, determined using a stereotaxic anatomical rat brain atlas (Paxinos and Watson, 2006). Signals were and amplified 1000-fold, low-pass filtered at 500 Hz and notch filtered at 50 Hz (EXT 10-2F, npi electronic GmbH, Germany). Data were digitized at 10 kHz by an analog to digital converter (CED 1401, CED Ltd., UK) and signal recording made was with Spike2 software (CED Ltd., UK). The signal was then downsampled to 1 kHz and exported to MatLab (Mathworks, Inc.) for further analysis.

### Experiment 1

*In vitro* Brain slices (*n* = 36) were prepared using the protocol described above and individual recordings made of spontaneous LFP activity from electrodes sited in LIII and LV (Figure 1B). These data were also integrated to produce an aggregate signal of deep and superficial M1 for comparison with MEG data. MEG Recordings were made from 13 healthy participants (7 M), with a mean age of 35.2 years, using the protocol described above. M1 was localized using SAM analysis and virtual electrode activity reconstructed during two periods of inactive rest (60 s). Data from individual LIII/LV *in vitro* electrode recordings and MEG virtual electrode M1 recordings were analyzed using Morlet-wavelet time-frequency analysis to identify the spontaneous oscillatory activity. The power-spectral-density (PSD) functions were determined for individual LIII and LV and the aggregate signal from *in vitro* M1 and compared to MEG M1. The relative contributions of deep and superficial layers were explored by weighting the ratio of LIII:LV power. The statistical difference between the spectral composition of the MEG signal and each *in vitro* weighting, were compared by computing the Kolmogorov–Smirnov (KS) D-statistic for each ratio using a custom script (Matlab, Mathworks, USA), as described previously (Baranauskas et al., 2012). The output of this is a measure of the statistical similarity between the oscillatory signatures of two timeseries.

### Experiment 2

*In vitro* brain slices (*n* = 17) were prepared using the protocol described above and individual recordings made of spontaneous LFP activity from electrodes sited in LIII and LV (Figure 1B). Ten minutes of control data were recorded, following which the GABA-A alpha-1 subunit modulator zolpidem (100 nM, Tocris Bioscience, Bristol, UK), was added to the aCSF perfusate. Following 45-min of incubation the spontaneous data were recorded from the same locations for a further 10-min.

Magnetoencephalography Recordings were made from eight healthy participants (6 M), with a mean age of 37.2 years, using the protocol described above. Participants underwent an initial control recording lasting 15-min, which included the functional M1 localizer task described above and two additional periods of inactive rest (60 s). Following the control recording, participants were administered zolpidem (0.05 mg/kg, Sanofi Aventis). Oral administration of 0.05–0.10 mg/kg produces a plasma concentration in the range 14–93 ng/ml (Olubodun et al., 2003; Greenblatt et al., 2006). As zolpidem is approximately 92% bound by plasma proteins (Salvà and Costa, 1995), these reports suggest that the effective concentration of zolpidem *in vivo* will be in the nanomolar range. Therefore, here we have used 100 nM as the comparative dose in our *in vitro* perfusion. Approximately 45-min following administration an identical recording to the control was made. Spontaneous M1 data from *in vitro* electrode recordings and MEG virtual electrode M1 recordings were analyzed using Morlet-wavelet time-frequency analysis and relative power change in the spontaneous oscillatory activity, in the pre and post zolpidem conditions, were visualized as normalized PSD. The power of the beta (15–30 Hz) and Mu (∼10 Hz) peaks was computed as an average of the normalized individual recordings and the statistical differences calculated using *t*-tests. The PSD functions were determined for individual LIII and LV and the aggregate signal from *in vitro* M1 and compared to MEG M1.

### Experiment 3

Experiment 3 used an *in vitro* approach only, in which slices (*n* = 12) were prepared using the same process described above. Slices were placed in the recording chamber and, following the acclimatization period, electrodes inserted into LIII and LV. Control data were recorded for 10-min, following which a custom made cutting device, comprising a ceramic blade, attached to a micro-manipulator (Narashige, Japan), was used to make an incision at the border of M1 and S1 (Figure 1B). All connection between M1 and S1 was severed over the course of 2 min (∼3.75 to 6 m/s), to avoid disturbing the ongoing recording or electrode positioning. Following incision, data were recorded for a further 10-min. The 60-s of data recorded pre and post cut from LIII and LV were analyzed using Morlet-wavelet time-frequency analysis and were visualized as normalized PSD. The statistical differences in the power of LV beta (15–30 Hz) and LIII mu (∼10 Hz) frequency peaks were calculated using *t*-tests.

## Results

### Experiment 1

**Figure 2:**
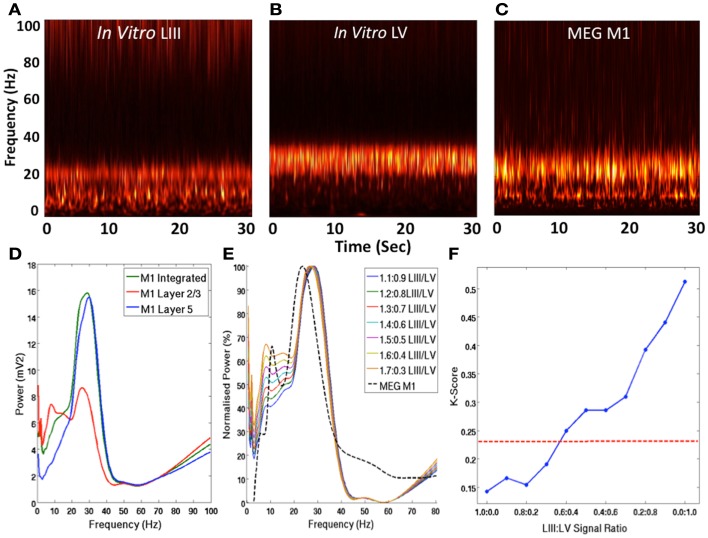
**Laminar Contribution to MEG M1 Oscillations**. **(A–C)** Show normalized group Morlet-wavelet time-frequency representations of the spontaneous activity in *in vitro* LIII, LV, and MEG M1 signals over 30 s. **(D)** Shows the PSD profiles of spontaneous LIII, LV, combined *in vitro* LIII + LV and MEG signals. **(E)** Shows a comparison of the group MEG PSD and the integrated *in vitro* signals with various weightings of LIII and LV. **(F)** Shows the Kolmogorov–Smirnov statistic for the comparison between MEG data and integrated *in vitro* data with various weightings. The red line shows the *p* = 0.05 threshold, below which signals are not statistically different.

Time-frequency analysis using the Morlet-wavelet approach reveals that in LIII of the *in vitro* recording, there were constant peaks at beta and mu frequencies (Figure 2A), whereas LV exhibited a peak only at beta frequency (Figure 2B). However, the MEG signal exhibited peaks at both beta and mu frequencies (Figure 2C), as expected from recording of the SMC (Pfurtscheller and Berghold, 1989). Integration of the signals from LIII and LV appear to present a signal that is more comparable with the MEG in terms of mu and beta amplitude (Figure 2D). Furthermore, adjustment of the weighting of these signals toward the contribution of LIII, appears to provide a better representation of the ration of mu and beta power observed in MEG (Figure 2E). Analysis of the statistical difference between these ratios using the Kolmogorov–Smirnov reveals that as signal is weighted toward LIII, the statistical difference between the signals is reduced (Figure 2F).

### Experiment 2

**Figure 3:**
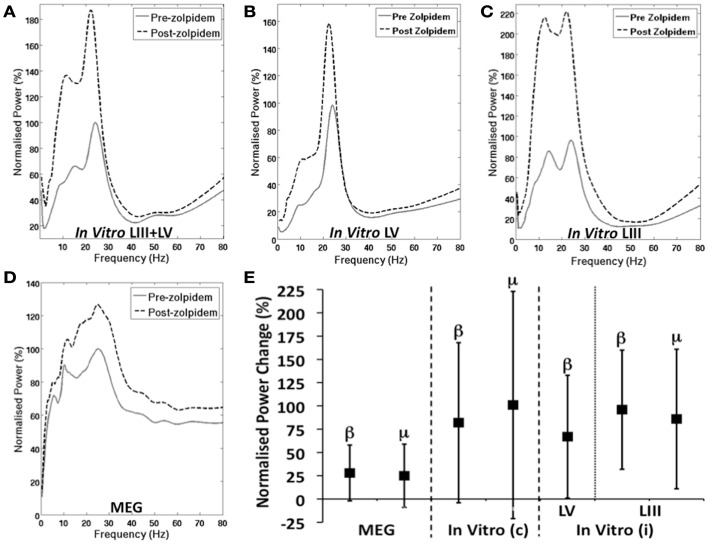
**GABAergic Modulation of M1 Oscillations**. **(A–D)** Shows the normalized group PSD profiles, before and after zolpidem. **(A)** Shows integrated layer 3 and layer 5 *in vitro* M1, **(B)** shows layer 5 only, **(C)** shows layer 3 only, and **(D)** shows MEG M1 before and after the administration of zolpidem. **(E)** Shows the mean increase (±SD) in power, following zolpidem, of mu and beta peaks in MEG (left), combined “*In vitro* (c)” layers 3 and 5 (middle) and independent “*In vitro* (i)” layers 3 and 5 (right).

The normalized PSD measures suggest an increase in power in the mu and beta range in both the integrated *in vitro* (Figure 3A) and MEG (Figure 3D) M1 recordings. Analysis of the peak power revealed significant increases in the mean beta power in MEG (28%, *t* = 2.5, *p* = 0.04) and integrated *in vitro* (82%, *t* = 2.21, *p* = 0.04). However, the increase in mean power of the mu rhythm was not significant in either MEG (25%, *t* = 1.84, *p* = 0.11) or integrated *in vitro* (85%, *t* = 1.89, *p* = 0.10) signals. Interestingly, when considering the individual laminae data, as expected there is a prominent beta peak in LV (Figure 3B), which shows a significant increase following zolpidem administration (67%, *t* = 2.2, *p* = 0.043). Similarly, there is a peak at beta frequency in LIII (Figure 3C) that shows an increase following zolpidem (96%, *t* = 2.47, *p* = 0.025). However, the mu peak present in LIII, also shows a significant increase following zolpidem (86%, *t* = 2.13, *p* = 0.049). The difference in mu between the integrated *in vitro* M1 and independent LIII, is a reflection of the low amplitude of the LIII signal compared to LV, consistent with previous observations (Yamawaki et al., 2008).

### Experiment 3

**Figure 4:**
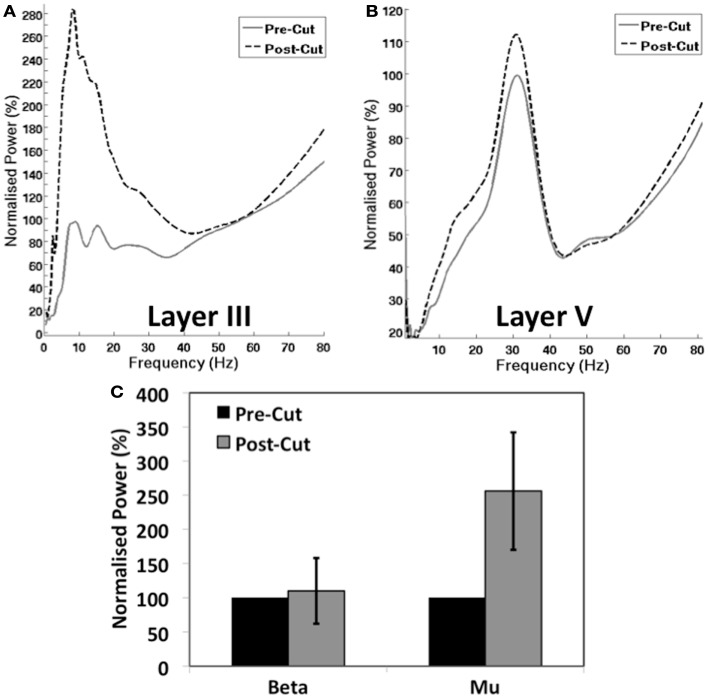
**Influence of Cortico-cortical Connectivity on M1 Oscillations**. **(A,B)** Show the normalized group PSD plots for layer 3 **(A)** and layer 5 **(B)** before and after the cut between M1 and S1 (see Figure 1B). **(C)** Shows the normalized mean group power of mu and beta peaks in M1 pre and post cut.

Analysis of the *in vitro* signal revealed that, following severance of the M1–S1 connection, there was a significant increase it the mean power of the mu rhythm in LIII (Figures 4A,C) (156%, *t* = 6.84, *p* = 0.006). However, a modest increase in the mean beta power in LV (Figures 4B,C) was non-significant (10%, *t* = 1.19, *p* = 0.26).

## Discussion

These findings suggest that MEG oscillations are an amalgamation of local field activity of layers III and V, consistent with previous predictions (Hamalainen, 1992; Murakami and Okada, 2006). In the case of the motor cortex, these data suggest that the mu and beta rhythms that are characteristic of observations of activity with electroencephalography (EEG) and MEG (Pfurtscheller and Berghold, 1989), are both represented within M1 in the *in vitro* signal. Based upon assessment of the GABAergic modulation of these signals, their response consistency supports the view that their generation is analogous between modalities. There are differences in the oscillatory profiles of the different laminae, which offers a mechanistic basis for differential modulation of these signals during function. This is further supported by the observation that connectivity between M1 and S1 produces profound effects on the superficial mu rhythm, but not deep beta rhythm. This observation may reflect a physiological basis, in the form of specific cortico-cortical projections, for the observations of independent pharmacological modulation of oscillations in the mu and beta range observed in somatosensory areas previously (Roopun et al., 2006).

### Composition of the neuroimaging signal

These experiments present evidence that the M1 exhibits multiple oscillations simultaneously. In addition to the well-characterized beta rhythm, we show for the first time, that an M1 mu rhythm also exists. These data contradict the view that these rhythms are generated by different cortical areas. Specifically, there are generators of mu in M1, which repudiates the concept of an exclusive S1 generator (Salmelin and Hari, 1994). However, it is important to consider the limitations involved with attributing MEG data to a single cortical area. Specifically, because of the close proximity of M1 and S1, it is quite possible that the virtual electrode profile of M1 will contain some contribution from S1. This is important when trying to attribute mu and beta contributions to deep and superficial M1.

Recent combined EEG and fMRI studies suggest that an inverse correlation between post-central BOLD amplitude and EEG rolandic mu, indicates that the generator of this rhythm lies in S1 (Ritter et al., 2009). While this multimodal approach offers a powerful combination for associating the functional coincidence of observations, it also highlights the difficulty in drawing conclusions from such parallel measurements. Specifically, we observe that connectivity between S1 and M1 has a profound effect on the amplitude of the mu rhythm (Figure 4A). Therefore, it is absolutely plausible that changes in neuronal network activity in S1, would modify the strength of connectivity between S1 and M1 and therefore mu power. Therefore, modulation of the BOLD signal by large metabolic changes in S1 could correspond to mu changes arising in M1. Furthermore, it is entirely possible that spontaneous mu rhythms are generated at multiple locations throughout the SMC, as is the case for beta rhythms (Roopun et al., 2006; Yamawaki et al., 2008). However, these data demonstrate that while various cortical rhythms may be under the influence of connectivity with other areas, these M1 beta and mu rhythms are not emergent properties of thalamo-cortical loops as suggested elsewhere (Rosanova et al., 2009).

### Mechanisms of neuroimaging signals

The data described here, suggest that the signals observed with MEG are a good reflection of the underlying activity in the cortex as measured *in vitro*. However, they also demonstrate that the composition of that signal is dependent upon the balance of contribution from the different laminae (Figure 2E), with an importance on the weighting toward superficial layers (Figure 2F), consistent with the principles of magnetic field decay in MEG (Hamalainen, 1992). This raises some important questions about the comparison of MEG with fMRI, as the BOLD signal is not subject to the same weighting of signals; all laminae have equal potential to contribute to the signal. This has important connotations for the attribution of BOLD changes to spatially coincident oscillatory changes. For example, where the tuning characteristics of signals in MEG are not consistent with those of BOLD (Muthukumaraswamy and Singh, 2008, 2009), this may be explained by differential laminar responses and the effect of signal weighting in MEG.

The comparable effects of GABAergic modulation in both models here (Figure 3), goes some way to increasing confidence in the analogous mechanisms of oscillatory generation. Specifically, this supports the view that motor cortex beta rhythms are under the control of previously described phasic entrainment by GABAergic interneurons (Whittington et al. (1995); Traub et al., 1996; Roopun et al., 2006; Yamawaki et al., 2008). This is consistent with previous MEG studies that describe a relationship between GABA concentration, or GABAergic modulation and beta in the motor cortex (Jensen et al., 2005; Muthukumaraswamy et al., 2009, 2012; Hall et al., 2010a,b, 2011; Gaetz et al., 2011). However, there are questions regarding the extent to which the sensorimotor mu rhythm is controlled by GABAergic mechanisms, refuted in previous MEG studies (Hall et al., 2010a). Given that recent studies demonstrate a direct relationship between GABA concentration and the characteristics of the hemodynamic response (Muthukumaraswamy and Singh, 2009; Donahue et al., 2010), an understanding of the proportion and profile of neural oscillations under GABAergic, and other, control is vital in order to obtain a comprehensive model of fMRI signals. However, while the concentration used in the *in vitro* experiment (100 nM) is believed to be of comparable physiological concentration to the 0.05-mg/kg dose used in the MEG experiment. It is important to bear in mind the variability of pharmacokinetics and drug metabolism in human participants are a limitation to the precise comparisons here.

### Cortical connectivity and neuroimaging signals

The data described here, demonstrate that the presence of oscillations in the motor cortex is not dependant upon connection with other cortical or sub-cortical locations. This suggests that neither beta nor mu rhythms *per se* are emergent properties of connectivity with locations such as the thalamus. However, the observation of increased power at mu frequency demonstrates that functional modulation of the power of an intrinsic oscillation can be the result of modulated connectivity with other areas. With regard to the mu rhythm, the data suggest that a reduction in the input to M1 produces an increase in mu power, from which it follows that an increase in the input to M1 may result in a reduction in mu power. A number of studies have demonstrated changes in the power of the mu rhythm during movement preparation, performance and recovery (Pfurtscheller and Berghold, 1989; Pfurtscheller et al., 1997; Leocani et al., 2001). These data would suggest that these phases are indicative of reduced connectivity between M1 and S1 or other areas during movement phases. Recent neuroimaging studies suggest that elevated mu prior to movement is a predictor of error in movement tasks (Mazaheri et al., 2009); which may be the result of a reduced connectivity during this phase. With regard to beta oscillatory power, connectivity with S1 did not influence the power in M1. This suggests that reductions in power during the preparation and execution phases of movement (Pfurtscheller and Berghold, 1989), are more likely the result of drive from cortical or sub-cortical areas other than S1; pre-motor areas appear to be of importance in this respect.

In addition, it is important to note that in the *in vitro* slice preparation, even before severing the connections between M1 and S1, no connections exist between this cortical area and other nodes of the motor network such as the basal ganglia. This highlights the important point, that the beta rhythm observed in motor cortex is not simply an emergent property of its connection with these structures. Conversely, it is unclear to what extent the nature of these oscillations is altered by the loss of connection with these structures. These questions could be addressed more comprehensively by the addition of *in vivo* measurements to those presented here.

## Conclusion

The present study demonstrates the commonalities between *in vitro* and MEG signals and provides strong support for the postulated source and mechanisms of signals recorded non-invasively in humans. However, it raises a number of important considerations that are particularly important when attempting to infer mechanisms of indirect measures of neuronal function, such as the fMRI BOLD response. Firstly, the fact that multiple oscillatory signatures arise within the same cortical area. Secondly, that these rhythms have independent mechanisms of generation and may, therefore, be modulated independently of one another. Finally, the role of connectivity between areas in the generation of oscillatory activity, should not be underestimated when interpreting the neural basis of non-invasively measured signals. In summary, while strong overlaps exist between MEG and fMRI signals, the subtleties of the intra- and inter-cortical constitution will influence this relationship.

## Conflict of Interest Statement

The authors declare that the research was conducted in the absence of any commercial or financial relationships that could be construed as a potential conflict of interest.
